# Multimorbidity and multi-disability among the elderly in residential care in India: the Hyderabad Ocular Morbidity in Elderly Study (HOMES)

**DOI:** 10.1038/s41598-022-15943-8

**Published:** 2022-07-11

**Authors:** Srinivas Marmamula, Thirupathi Reddy Kumbham, Rahul Shidhaye, Satya Brahmanandam Modepalli, Navya Rekha Barrenkala, Ratnakar Yellapragada, Jill Keeffe

**Affiliations:** 1 Allen Foster Community Eye Health Research Centre, Gullapalli Pratibha Rao International Centre for Advancement of Rural Eye care, L V Prasad Eye Institute, Hyderabad, 500034 India; 2 Brien Holden Institute of Optometry and Vision Science, L V Prasad Eye Institute, Hyderabad, India; 3 Department of Biotechnology, Wellcome Trust India Alliance, L V Prasad Eye Institute, Hyderabad, India; 4 School of Optometry and Vision Science, University of New South Wales, Sydney, Australia; 5 Pravara Institute of Medical Sciences, Loni, Ahmednagar, Maharashtra, India

**Keywords:** Diseases, Health care, Medical research

## Abstract

We report prevalence and risk factors for multimorbidity and multi-disability among elderly people in residential care in the Hyderabad region in South India. In total, 1182 elderly (aged ≥ 60) participants were examined in 41 homes for the aged centres. Detailed interviews were conducted by trained personnel to collect personal and demographic information. A questionnaire was used to assess the history of non-communicable diseases and Washington Disability Questionnaire (WDQ) was administered to assess disabilities. The mean age of the participants was 75.0 years (SD 8.8 years; range: 60–108 years), 35.4% were men, 20.3% had no formal education, 60.7% had school education and 19% had higher education. The prevalence of multimorbidity was 37.6% (95% CI: 34.8–40.4). Prevalence of multi-disability was 23.6% (95% CI: 21.2–26.3; n = 270). In total, 857 (72.5%) participants reported using at least one medication for NCDs. Over a third of the elderly in residential care had multimorbidity, and a quarter of them had multi-disability. A holistic health care system that comprises health and wellness coupled with rehabilitation to address disabilities is needed to achieve healthy aging in elderly in homes for the aged in India.

## Introduction

Aging is the hallmark of the twentieth century. An increase in the size of the elderly population has resulted in significant changes in the demographic profile in most countries. In India, individuals aged 60 years and older are considered elderly. From being less than 10% in 2010, the proportion of elderly in the population is expected to double by 2050^1,2^. Also, the number of elderly individuals living separately from their children in the same or different locations, either alone or with their spouses, have gone up due to societal changes^3,4^. The rise in the number of homes for the aged, especially in urban and suburban areas in India, is another spinoff^3^.

Globally over a billion people are estimated to have disabilities^5^. Aging is often associated with a higher prevalence of non-communicable diseases (NCDs) and disabilities^5,6^. A high prevalence of hypertension, diabetes and heart diseases have been reported in the community-dwelling elderly population in South India and also in the general population in India^7–9^. The 2011 census in India revealed that 5.1% of the elderly had various kinds of disabilities^10^. A more recent population-based study from the state of Telangana reported that every fifth elderly had at least one disability and every third individual had at least one NCD^11^. Other community-based studies conducted in India have shown a high prevalence of disabilities and NCDs in the community-dwelling elderly population^12–15^. Also, a high prevalence of visual impairment has been reported in the elderly in South India^16^. A few studies have also highlighted the adverse effect that various kinds of disabilities have on the quality of life of the elderly and their wellbeing^17^. Multimorbidity, defined as two or more systemic health issues (NCDs) in an individual, is common among the elderly, with a prevalence ranging from 24% to as high as 83%^18,19^. The adverse effect of multimorbidity in the elderly has been widely reported. However, such data on elderly people in residential care (homes for the aged) is not available^20–24^.

The Hyderabad Ocular Morbidity in Elderly Study (HOMES) was carried out in homes for the aged centres located in and around Hyderabad in the South Indian state of Telangana^25^. It was aimed to broaden the understanding of the burden of vision loss, its causes and associated risk factors among the elderly in residential care^25^. The HOMES study reported that 30.1% of the elderly had vision impairment^26^. The evaluation of HOMES participants also included collecting detailed information on NCDs including, diabetes, hypertension, heart disease, muscle-skeletal disorders and other conditions. The information on disabilities was collected using the Washington Disability Questionnaire^27,28^. In this paper, we report on the prevalence and risk factors for multimorbidity and multi-disability in the elderly in residential care.

## Methods

### Ethics approval

The study protocol was approved by the Institutional Review Board of the Hyderabad Eye Research Foundation, L V Prasad Eye Institute, Hyderabad. Each participant provided written informed consent for their participation in the study. The study was carried out between 2017 and 2019 in accordance with the Declaration of Helsinki.

### Sample selection

This study was nested in a study that aimed to understand the prevalence, causes and risk factors of visual impairment. Based on an anticipated prevalence of visual impairment of 15%, a precision of 20% prevalence (± 3%), a non-response rate of 25%, and a design effect of 1.4 to account for clustering, a sample size of 916 individuals was required. This sample is also sufficient for estimation of the prevalence of systemic conditions (NCDs) and disabilities as the prevalence of these conditions is higher than the prevalence of visual impairment.

### Study population

The study included all the 46 homes that agreed to participate (including the five homes selected for the pilot study) from the 76 homes for the aged in the Hyderabad region in Telangana state. About a third of the homes did not consent to participate in the study. The main reasons stated for non-participation were, (a) preference to visit the eye care providers when needed and not to burden the elderly with regular eye examinations as part of the project and (b) no consent and/or non-approval from the family members of the elderly for participation in the study. In addition, six homes agreed to participate in the study, but space was not available for setting up the clinic for assessment.

Residents aged ≥ 60 years, who agreed to participate and were living in these homes for at least one month at the time of enumeration, were included. We have reported the description of these homes, participants' characteristics and the study protocol in our previous publications^25,26,29^. In brief, the homes were classified as (1) private homes where the individuals or their kin pay a monthly or annual user fee; (2) aided/partially subsidized homes where the individuals or their kin pay a part of the user fee, and the rest of the amount is met by philanthropic support or through other funding sources; (3) free homes where individuals need not pay any user fee as they are supported through external funding.

As described in our previous publications, all the participants underwent a detailed interview by trained investigators^25^. It involved recording their personal and socio-demographic information, ocular and systemic history (including information on diabetes, hypertension, heart disease and musculoskeletal issues), lifestyle factors such as smoking and alcohol consumption. For those participants who reported systemic condition/s, the duration of the diagnosis of the condition/s and current medication/s were asked and documented. Additionally, the details regarding the current medication/s taken by the residents (after seeing the supplies), their cost and the frequency of each medication/s were recorded. Multimorbidity is defined as reporting two or more NCDs in an individual. Polypharmacy was defined as the use of five or more medication daily^30^.

### Study tools

The Washington Group (WG) developed a brief questionnaire [Washington Disability Questionnaire (WDQ)] to facilitate quick and comparable data collection on disabilities in population-based studies and the population census^27,28^. These are the most commonly used questionnaires to estimate the prevalence of disabilities. These WG questionnaires are used in over 100 national population censuses to date^27^. They are also recommended by United Nations and other agencies as indicators for Sustainable Development Goals (SDG) and United Nations Convention on the Rights of People with Disabilities. The WG questionnaires were also used in surveys in India (including the state of Telangana where the current study was conducted) and in other developing countries^31^.

The Washington Disability Questionnaire was used to assess disabilities in this study^25^. It was administered to the participants in their local language (Telugu or Hindi) before the eye examination, and included the following questions: (a) Do you have difficulty seeing, despite wearing glasses? (b) Do you have difficulty hearing, despite using a hearing aid? (c) Do you have difficulty walking or climbing steps? (d) Do you have difficulty remembering or concentrating? (e) Do you have difficulty (with self-care such as) cleaning yourself or dressing? (f) Using your usual (customary) language, do you have difficulty communicating, for example, understanding or being understood? Each of these questions had four options based on severity, such as (1) Not at all, (2) Some difficulty, (3) Lot of difficulties and (4) Cannot do at all. ‘Not at all’ and ‘Some difficulty’ were considered as no disability. ‘Lot of difficulties’ and ‘Cannot do at all’ were considered as a disability^11^ . Multi-disability is defined as reporting two or more disabilities in an individual.

### Covariates

Independent variables included age group (60–69, 70–79 and ≥ 80 years), gender (male or female), education (higher education/graduation and above), school education only (1–12 years of education), or no education, marital status (married, widowed/separated, single), type of home (free, subsidized or private), smoking (never smokers and ever smokers, including current and past smoker) and alcohol consumption (no alcohol and ever alcohol, including current and past alcohol consumption).

### Data management

Data analysis was conducted using Stata Statistical Software for Windows, version 14 (StataCorp, College Station, TX)^32^. Prevalence estimates were calculated and presented with 95% Confidence Intervals (CI). Logistic regression models were used to examine the strength of association between multimorbidity and the potential risk factors. In multiple logistic regression analysis, multimorbidity was used as an outcome variable and its association with other sociodemographic covariates (age, gender, level of education, marital status, type of home) was assessed. In the multivariable regression model, covariates were included based on published literature, and were entered into the model at a time. A similar analysis was also conducted for multi-disability. The Hosmer–Lemeshow goodness of fit test was used to assess the model fit. Variance Inflation Factors (VIF) were used to test collinearity between the covariates after fitting a multiple regression model. The adjusted odds ratios (OR) with 95% confidence intervals (CI) were presented. For all analyses, the statistical significance was assessed at the conventional level of p less than 0.05 (two-tailed). However, the exact p values were reported.

## Results

### Characteristics of the participants

Fifteen hundred and thirteen residents were enumerated from 41 homes, of which 1182 (78.1%) were interviewed and examined in the makeshift eye clinics set up in the homes. The demographic profile of the study participants is presented in our previous publications^25,26,29^. In short, 179 (11.8%) participants were not available for examination even after two attempts and 152 (10.1%) refused to undergo eye examinations. The mean age (p = 0.05) and gender (p = 0.31) distribution of the participants who were examined and non-examined were similar. The mean age of examined participants was 75.0 years (SD 8.8 years; range: 60–108 years), 35.4% (n = 418) were men, 20.3% (n = 240) had no formal education, 60.7% (n = 717) had school education and 19% (n = 225) had higher education. In all, 501 (42.4%) participants were living in paid/private homes, 419 (41.5%) were living in subsidised homes, and 190 (16.6%) were living in free homes. Data on Body Mass Index (BMI) was available for 929/1,182 (78.6%) participants, of which 463 (49.8%) were normal/underweight, 310 (33.4%) were overweight, and 156 (16.8%) were obese.

### Multimorbidity

**Figure 1:**
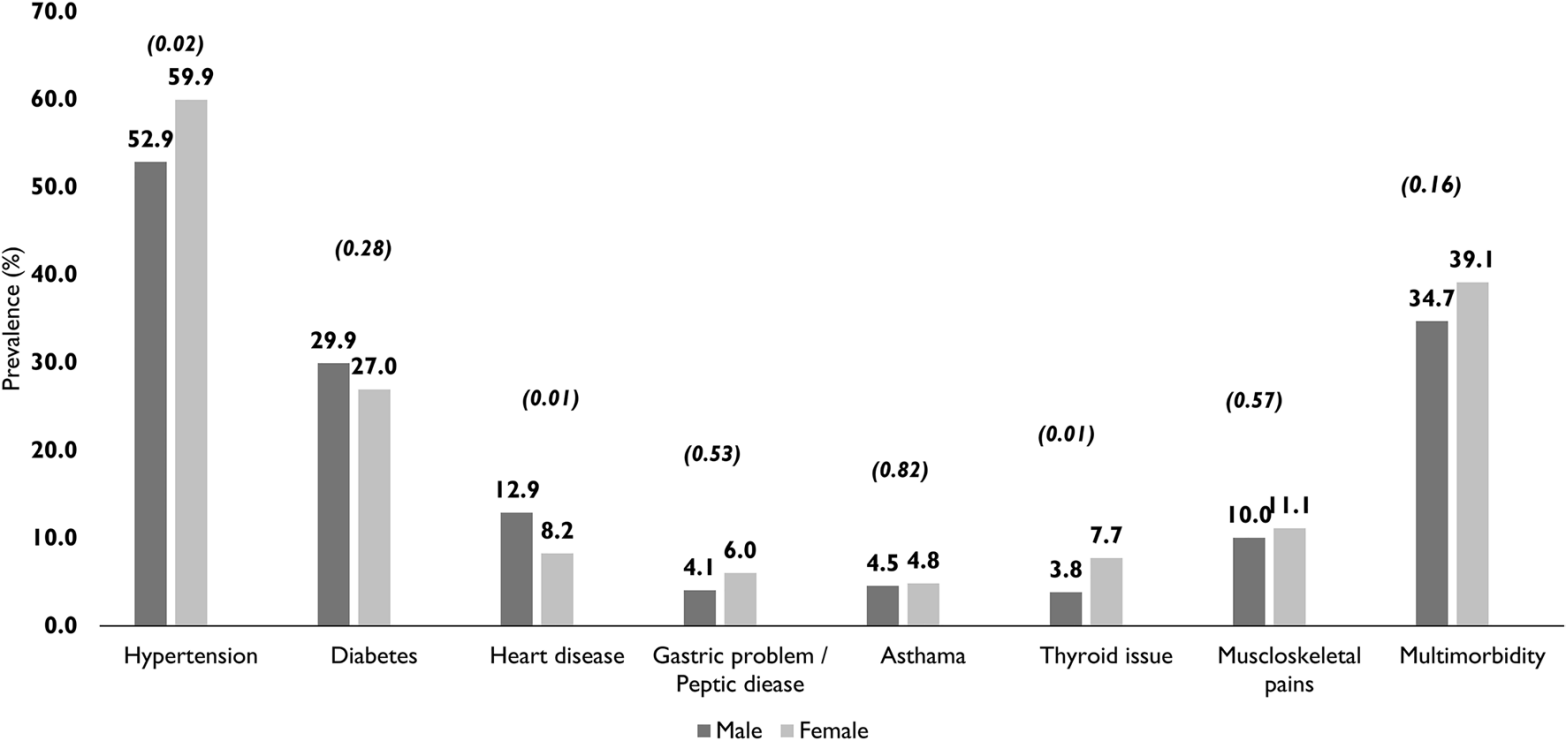
Prevalence of non-communicable diseases and multimorbidity stratified by gender (n = 1182); Statistical significance is shown in parenthesis (Chi squared test).

The prevalence of multimorbidity was 37.6% (95% CI: 34.8–40.4; n = 444). Overall, 836 (70.7%; 95% CI: 68.0–73.3) participants reported at least one NCD. The prevalence of multimorbidity stratified by personal and socio-demographic factors is shown in Table 1. Hypertension was the most common NCD (57.4%; 95% CI: 54.5–60.3; n = 678) followed by diabetes (28.0%; 95% CI: 25.4–30.6; n = 331), musculoskeletal pains (10.7%; 95% CI: 9.0–12.6; n = 127) and heart disease (9.9%; 95% CI: 8.2–11.7; n = 117) (Fig. 1).

**Table 1:** Association of multimorbidity (≥ 2 conditions) with sociodemographic and other characteristics (multiple logistic regression analysis).

	Total in the sample (n)	Participants with multimorbidity (n)	Prevalence (95% CI)	Crude odds ratio (95% CI)^a^	P value	Adjusted Odds ratio (95% CI)^b^	P value
**Age group (years)**							
60–69	329	113	34.3 (29.2–39.7)	Reference		Reference	
70–79	453	188	41.5 (36.9–46.2)	1.36 (1.01–1.82)	0.043	1.37 (1.01–1.86)	0.04
80 and above	400	143	35.8 (31.0–40.7)	1.06 (0.78–1.44)	0.70	1.13 (0.82–1.56)	0.46
**Gender**							
Male	418	145	34.7 (30.1–39.5)	Reference		Reference	
Female	764	299	39.1 (35.6–42.7)	1.21 (0.94–1.55)	0.131	1.40 (0.98–1.99)	0.07
**Education level**							
Higher education	225	82	36.4 (30.1–43.1)	Reference		Reference	
School education	717	282	39.3 (35.7–43.0)	1.13 (0.83–1.54)	0.44	1.15 (0.82–1.620)	0.8
No education	240	80	33.3 (27.4–39.7)	0.87 (0.60–1.28)	0.48	1.06 (0.68–1.64)	0.26
**Type of home**							
Free home	190	51	26.8 (20.7–33.7)	Reference		Reference	
Subsidised home	491	196	39.9 (35.4–44.4)	1.81 (1.25–2.62)	< 0.01	1.73 (1.18–2.53)	0.01
Paid homes	510	197	38.6 (34.4–43.0)	1.77 (1.22–2.55)	< 0.01	1.79 (1.20–2.67)	< 0.01
**Smoking status**							
Never	976	364	37.3 (34.2–40.3)	Reference		Reference	
Current/past	206	80	38.8 (32.1–45.9)	1.07 (0.78–1.45)	0.678	1.59 (1.04–2.43)	0.03
**Alcohol consumption**							
Never	971	371	38.2 (35.1–41.3)	Reference		Reference	
Current/past	211	73	34.6 (28.2–41.4)	0.86 (0.63–1.17)	0.327	0.87 (0.60–1.28)	0.49
**Body mass index (BMI)**							
Normal/underweight (< 18.5–25)	463	149	32.2 (27.9–36.6)	Reference		Reference	
Overweight (> 25–30)	310	147	47.4 (41.7–52.1)	1.90 (1.41–2.56)	< 0.01	1.86 (1.37–2.52)	< 0.01
Obese (> 30)	156	73	46.8 (38.8–54.9)	1.85 (1.28–2.68)	< 0.01	1.71 (1.16–2.51)	0.01
Missing data	253	75	29.6 (24.1–35.7)	0.89 (0.63–1.24)	0.49	0.84 (0.60–1.19)	0.33
**Total**	**1182**	**444**	**37.6 (34.8–40.1)**				

^a^Based on simple logistic regression with multimorbidity as the outcome variable.

^b^Based on multiple logistic regression with multimorbidity as the outcome variable and the predictors entered at the same time.

On simple logistic regression analysis, multimorbidity was associated with 70–79 years age group, type of residence, and BMI. On multiple logistic regression analysis, the multimorbidity was associated with older age groups. In comparison with those aged 60–69 years, the odds of having multimorbidity were higher for those aged 70–79 years (OR:1.37; 95% CI: 1.01–1.86) and 80 years and older age groups (OR: 1.13; 95% CI: 0.82–1.56). In comparison to those elderly who resided in ‘free’ homes, the odds for multimorbidity were higher for those residing in subsidised’ (OR: 1.73; 95% CI: 1.18–2.53) and ‘private homes’ (OR:1.79; 95% CI: 1.20–2.67). Compared to the elderly who had normal BMI, the odds for multimorbidity were higher among those who were overweight (OR: 1.86; 95% CI: 1.37–2.52) and obese (95% CI: 1.71; 95% CI: 1.16–2.51). Gender, level of education, smoking, and alcohol consumption were not associated with multi-morbidity. (Table 1).

Table 2 shows the stratification of numbers of NCDs among the participants (Table 2).

**Table 2:** Prevalence of number of non-communicable diseases with 95% confidence intervals.

	Number of participants	Prevalence (95% CI)
No NCD	346	29. 3 (26.7–31.9)
One NCD	392	33. 3 (30.5–36.0)
Two NCDs	309	26.1 (23.7–28.7)
Three NCDs	108	9.1 (7.6–10.9)
Four or more NCDs	27	2.3 (1.5–3.3)
**Total**	**1182**	

### Multi-disability

**Figure 2:**
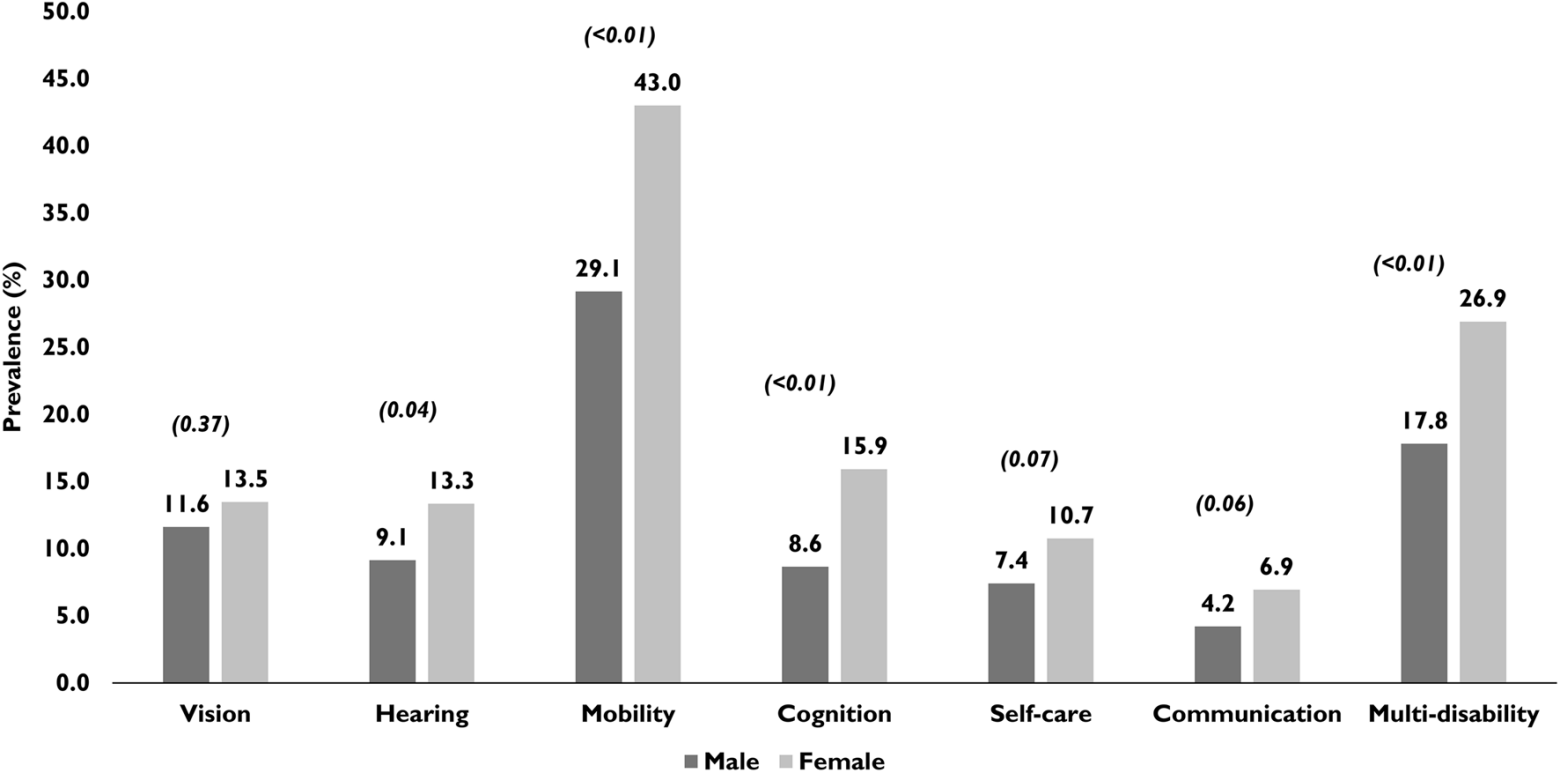
Prevalence of disabilities and multi-disability stratified by gender (n = 1140); statistical significance is shown in parenthesis (Chi squared test).

Data on disabilities were available for 1,140/1,182 (96.4%) participants. The prevalence of multi-disability was 23.6% (95% CI: 21.2–26.3; n = 270). Half (50.0%) of the elderly residents had at least one disability (95% CI: 47.0–52.9; n = 570). In terms of the number of disabilities in an individual, 300 (26.3%) reported one disability, 153 (13.4%) reported two disabilities and 117 (10.3%) reported three or more disabilities. Mobility (38.1%; 95% CI: 35.2–41.0) was the most commonly reported disability followed by cognition (13.3%; 95% CI: 11.4–15.4), vision (12.8%; 95% CI: 10.9–14.9), hearing (11.8%; 95% CI: 10.0–13.9), self-care (9.6%; 95% CI: 7.9–11.5) and communication (6.0%; 95%: 4.7–7.5) (Fig. 2).

On simple logistic regression analysis, multi-disability was associated with older age groups, lower levels of education and higher BMI categories. On multiple logistic regression analysis, multi-disability was associated with 80 years and older age group (OR:2.50; 95% CI: 1.64–3.33). Those elderly who had no education had significantly higher odds (OR: 2.60; 95% CI: 1.31–5.15) for multi-disability compared to those who had higher education. Though odds for multi-disability were higher among those with school education, it was not statistically significant (p = 0.99). Multi-disability was associated with the type of home with individuals living in subsidised/aided homes having higher odds (OR:1.60; 95% CI: 1.06–2.57) compared to those living in paid homes. Gender, alcohol, and smoking status and BMI were not associated with multi-disability (Table 3).

**Table 3:** Association of multi-disability (≥ 2 conditions) with sociodemographic and other characteristics (multiple logistic regression analysis) ;

	Total in the sample (n)	Participants with multi-disability (n)	Prevalence (95% CI)	Crude odds ratio (95% CI)^a^	P value	Adjusted odds ratio (95% CI)^b^	p value
	**n**		**(n = 1140)**				
**Age group (years)**							
60–69	323	52	16.1 (12.3–20.6)	Reference		Reference	
70–79	445	93	20.9 (17.2–25.0)	1.38 (0.95–2.00)	0.094	1.41 (0.93–2.12)	0.105
80 and above	372	125	33.6 (28.8–38.7)	2.64 (1.83–3.80)	< 0.01	2.50 (1.64–3.77)	< 0.01
**Gender**							
Male	405	72	17.8 (14.2–21.8)	Reference		Reference	
Female	735	198	26.9 (23.8–30.3)	1.71 (1.26–2.31)	< 0.01	1.48 (0.93–2.34)	0.094
**Education level**							
Higher education	223	31	13.9 (9.6–19.1)	Reference		Reference	
School education	696	161	23.1 (20.0–26.4)	1.86 (1.23–2.83)	< 0.01	1.35 (0.75–2.45)	0.321
No schooling	221	78	35.3 (29.0–42.0	0.30 (0.18–0.47)	< 0.01	2.60 (1.31–5.51)	0.006
**Type of homes**							
Free home	184	39	21.2 (15.5–27.8)	Reference		Reference	
Subsidised home	473	122	25.8 (21.9–30.0)	1.29 (0.86–1.95)	0.22	1.60 (1.06–2.57)	0.051
Paid homes	483	109	22.6 (18.9–26.6)	1.1 (0.71–1.64)	0.7	1.0 (0.61–1.64)	0.997
**Smoking status**							
Never	939	234	24.9 (22.2–27.8)	Reference		Reference	
Past/current	201	36	17.9 (12.9–23.9)	0.66 (0.45–0.97)	0.035	0.97 (0.54–1.70)	0.923
**Alcohol status**							
Never	935	228	24.2 (21.7–27.3)	Reference		Reference	
Past/current	205	42	20.5 (15.2–26.7)	0.80 (0.55–1.16)	0.235	1.12 (0.68–1.83)	0.66
**Body mass index (BMI)**							
Normal/underweight (< 18.5–25)	457	75	16.4 (13.1–20.1)	Reference		Reference	
Overweight (> 25–30)	310	50	16.1 (12.2–20.7)	0.98 (0.66–1.45)	0.917	1.08 (0.71–1.60)	0.752
Obese (> 30)	156	18	11.5 (7.0–17.6)	0.66 (0.38–1.15)	0.145	0.70 (0.40–1.24)	0.223
Missing data	217	127	58.5 (51.7–65.1)	7.18 (5.0–10.40)	< 0.01	7.54 (5.10–11.17)	< 0.01
**Total**	**1140**	**270**	**23.7 (21.2–26.3)**				

^a^Based on simple logistic regression with multi-morbidity as the outcome variable.

^b^Based on multiple logistic regression with multi-morbidity as the outcome and all the predictors entered at the same time.

Table 4 shows the stratification of number of disabilities in the study participants (Table 4).

**Table 4:** Prevalence of number of non-communicable diseases with 95% confidence intervals.

	Number of participants	Prevalence (95% CI)
No disability	570	50.0 (47.1–53.9)
One disability	300	26.3 (23.8–29.0)
Two disabilities	153	13.4 (11.5–5.5)
Three disabilities	61	0.54 (4.1–0.7)
Four or more disabilities	56	4.9 (3.7–6.3)
Total	1140	

### Current medication and assistive devices usage

In total, 840/1,182 (71.1%) of the participants reported using at least one medication. Among these, 261 (31.3%) participants were on one medication, 386 (46.2%) were on 2–4 medications. Polypharmacy (5 or more medications) was reported by 88 (22.5%) participants. In terms of assistive devices being used, spectacles for vision (57.1%) were the most common devices, followed by devices for walking (29.7%), including 15 people who were using a wheelchair for mobility, and hearing aids (3.1%). One person reported using a hand magnifier for near work. Approximately 70% (n = 823) of the elderly were using at least one assistive device (69.6%; 95%CI: 66.9–72.2). Among those using assistive devices, 70.6% (n = 581) were using one device, 27.7% (n = 228) were using two devices, and only 1.7% (n = 14) were using three devices.

On multiple logistic regression analysis, the use of an assistive device was associated with older age with odds of 1.83 (95% CI:1.35–2.49) for those aged 70–79 years and 2.22 (95% CI: 1.60–3.09) for those aged 80 years and older. Those elderly who had higher levels of education were more likely to use an assistive device. Higher odds were also found for those with school education (OR:1.70; 95% CI:1.24–2.34) and higher education (OR:2.14; 95% CI:1.35–3.38) compared to those without any education. The use of assistive devices was not associated either with gender or type of home (Table 5).

**Table 5:** Association of at least one assistive device used with sociodemographic characteristics (multiple logistic regression analysis).

	Total in the sample (n)	Participants using at least one assistive device (n)	Prevalence (95% CI)	Adjusted odds ratio (95% CI)	P-value
**Age group (years)**					
60–69	329	191	58.1 (52.5–63.4)	Reference	
70–79	453	327	71.2 (67.8–76.3)	1.83 (1.35–2.49)	< 0.01
80 and above	400	305	76.2 (71.2–8.03)	2.22 (1.60–3.09)	< 0.01
**Gender**					
Male	418	313	73.9 (70.4–79.0)	Reference	
Female	764	510	66.7 (63.3–70.1)	0.82 (0.61–1.10)	0.19
**Education**					
No education	240	138	57.5 (51.0–63.8)	Reference	
School education	717	511	71.2 (67.8–74.6)	1.70 (1.24–2.34)	< 0.01
Higher education	225	174	77.3 (71.3–82.6)	2.14 (1.35–3.38)	< 0.01
**Type of homes**					
Free home	190	109	57.3 (50.0–64.50)	Reference	
Partially paid/aided	491	347	70.7 (66.4–74.7)	1.41 (0.98–2.02)	0.07
Completely paid/private homes	501	367	73.2 (69.1–77.1)	1.36 (0.93–1.98)	0.11
**Total**	**1182**	**823**	**69.6 (66.9–72.2)**		

## Discussion

One out of every three elderly in residential care had multimorbidity, and a quarter of them had multi-disability. Over two-thirds of the elderly had at least one NCD, and half of them had at least one disability. Also, close to 73% of them were on at least one medication for their systemic health. Close to 70% of the elderly were using at least one assistive device, spectacles being the most commonly used device. Only 3% of the elderly were using hearing aids despite hearing impairment being one of the common disabilities. A large burden of NCDs and disabilities was also reported in a community-based sample of the elderly population in Telangana state using a similar protocol^11^.

Multimorbidity was associated with older age groups, similar to other studies in India and other regions^33,34^. The odds were higher among those living in subsidized (aided) and private homes than those staying in free homes. The prevalence of multimorbidity was recorded based on self-reporting. It is possible that those living in private and subsidised homes had better access to care or were more health conscious. Thus, they sought health care and were more aware of their health issues. And, with more resources at their disposal, they tended to live in subsidized and private homes.

Diabetes and hypertension are public health challenges in India and highly prevalent among elderly individuals in the community^7,8,35,36^. Both diabetes and hypertension are known to be associated with irreversible vision loss. Hence, they are of special interest to the eye care community. From the service delivery aspect, these conditions need continuity of care to ensure that they are in control and do not lead to complications and subsequent disabilities^37–39^.

In our study population, similar to the population-based studies, we found a high prevalence of mobility issues, vision loss and hearing impairment^11^. A population-based study using a similar examination protocol in the same state has reported that one-fifth of the elderly had at least one disability, and over a third of them had at least one NCD^11^. Some studies have reported a large regional variation in the burden of disabilities within the same states ranging from 23.4 to 53.6% in Uttar Pradesh ^40,41^ and from 20.6 to 68% in Tamil Nadu^42^. The study conducted in Tamil Nadu reported visual disability as more common compared to mobility^42^.

Two other studies, one in Odisha and one in Rajasthan, have reported the prevalence of cognitive impairment as 25% and 51%, respectively—much higher than what we found in our study in Telangana^43,44^. The variability reported in the burden is due to the varying methods adopted in these studies and the study participants. Moreover, the results from these community-based studies are not comparable with our study because of difference in lifestyle and other socio-demographic factors of the participants.

Disabilities were associated with older age with significantly higher odds among those aged 80 years and older. Interestingly, disability was not associated with gender and BMI. After adjusting for covariates, those with no education and residing in free homes had higher odds for disability. It is suggestive of their poor access and limited financial resources to seek care. Disability is often related to the inability of an individual to perform a particular task as perceived by an individual. On the other hand, NCDs such as hypertension and diabetes are often based on biological parameters and can result in disabilities as a complication. Those with higher levels of education were probably better at managing their systemic conditions resulting in lower odds of disabilities than those with no education. They were also more likely to use assistive devices.

Providing quality and holistic health care and rehabilitation are public health challenges. And in India, this challenge is expected to compound in future years with the demographic transition. But without the implementation of appropriate health policies—healthy and happy aging—cannot be achieved. The governments, especially in developing countries such as India, are developing socio-economic schemes and health welfare policies and programmes focusing on the elderly^45^.

The Government of India launched the National Programme for Health Care of the Elderly (NPHCE) in 2010 to create a health care framework for the elderly to provide accessible, affordable, and comprehensive health care^17,46^. It also includes financial support in terms of a monthly pension for the elderly. The National Health Policy of 2017 aims for universal and affordable health care for all. There is a need to study the impact of these initiatives and replicate them with a special focus on the elderly in residential care^47^. An integrated approach that includes physical and mental health, social services, lifestyle, and community institutions is recommended^48^.

In India, a few non-government organizations have been focussing on elderly health and wellness^49^. To ensure continuity of care, there is a need to make home-based care available to these vulnerable groups by using innovative approaches such as telehealth and other similar models. Also, there is a need for comprehensive elderly-centric health care covering all aspects of the elderly including, health and wellness, yoga and meditation for mental and physical wellbeing, rehabilitation, and assistive devices for disabilities. Novel home-based eye care initiatives for the elderly eye care are also being pilot tested in India. If found successful, these initiatives can be expanded to incorporate preliminary screening for NCDs and disabilities in the elderly with appropriate referrals and eye health could thus become an entry point for holistic health care for the elderly^50^.

We reported on multimorbidity and multi-disability among the elderly in residential care as a part of a larger eye health study for the first time in India. The study also assessed the use of assistive devices. Our results can also be generalized to the elderly in residential care in other urban areas in India. We used a questionnaire-based approach and self-report from the elderly and did not do the objective assessment of the health conditions. It could have resulted in a potential bias. Also, as we have used a cross-sectional study design, the association between the outcome and other variables cannot be firmly established. Despite these limitations, our study has provided valuable insights on disability and the systemic health situation of the elderly in residential care. The insights provide a good base for developing elderly centric services to contribute to healthy ageing in India.

## Data Availability

The datasets generated during the study are not publicly available as further data analysis is being carried out. The data can be made available from the corresponding author on a reasonable request.
